# Supplementary material for: Content-rich biological network constructed by mining PubMed abstracts
Source: BMC Bioinformatics. 2004 Oct 8;5:147. doi: 10.1186/1471-2105-5-147 (PMC528731; doi:10.1186/1471-2105-5-147)
Supplement: Additional File 2 — The original results of the above study (non-essential files are deleted to keep the file size under the limit set by BMC bioinformatics). [file 1471-2105-5-147-S2.bz2 › chilibotAdditionalFile2/dip40/488ID9486651E10603/html/CXCL13_BLR1.html]

 


 **CXCL13** and **BLR1** 
  
Found 38 abstracts in PubMed, retrieved 38.  
 

 What does Google say? 
 PDF only 
| .edu only 

---

**Interactive relationship** (e.g. stimulation, inhibition, etc)

**Neutral relationship**- FL cells expressed CXCR5  [ **BLR1** ]  and migrated in response to  **CXCL13** .  Ref: 12406091 Br J Haematol, 2002
- CD3 CD4 IL 7Ralphahi cells express the chemokine receptor CXCR5  [ **BLR1** ]  and might be attracted by its ligand  **CXCL13** , which is secreted by mesenchymal cells.  Ref: 12732661 J Exp Med, 2003
- A migratory population of skin derived dendritic cells expresses CXCR5  [ **BLR1** ] , responds to B lymphocyte chemoattractant  [ **CXCL13** ]  in vitro, and co localizes to B cell zones in lymph nodes in vivo.  Ref: 11069061 Eur J Immunol, 2000
- The lymphoid population expresses CXCR5  [ **BLR1** ]  and has a strong chemotactic response to B lymphocyte chemoattractant  [ **CXCL13** ]  BLC  [ **CXCL13** ] .  Ref: 11238592 J Exp Med, 2001
- The discovery that many follicular stromal cells make B lymphocyte chemoattractant  [ **CXCL13** ]  BLC  [ **CXCL13** ] , a CXC chemokine that attracts CXCR5  [ **BLR1** ]  cells, provides a basis for understanding how motile B cells come into contact with stationary FDCs.  Ref: 11043777 Immunol Rev, 2000
- Skin derived migratory dendritic cells DC, in contrast to bone marrow derived DC BMDC, express CXCR5  [ **BLR1** ] , respond to the chemokine CXC ligand 13  **CXCL13**  in vitro, and are capable of migrating to B cell zones BCZ in lymph nodes LN in vivo.  Ref: 11994462 J Immunol, 2002
- When tested directly ex vivo, CXCR5  [ **BLR1** ]  hi T cells exhibited a vigorous chemotactic response to BLC  [ **CXCL13** ] .  Ref: 10523610 J Exp Med, 1999
- B cell attracting chemokine 1  [ **CXCL13** ]  BCA 1  [ **CXCL13** ]  responses correlate with CXC chemokine receptor CXCR 5  [ **BLR1** ]  expression, are first displayed by a pro B cell subset, are lost in pre B cells, and then are regained just before and after egress from the marrow.  Ref: 10770798 J Exp Med, 2000
- CXCR5  [ **BLR1** ]  was detected by flow cytometry on transduced cells, which responded to  **CXCL13**  in vitro in chemotaxis assays 3 fold over nontransduced BMDC, p <.  Ref: 11994462 J Immunol, 2002
- BCA 1  [ **CXCL13** ]  was chemically synthesized and tested for activity on murine pre B cells 300 19 transfected with  **BLR1**  and on human blood B lymphocytes.  Ref: 9463416 J Exp Med, 1998
- In low grade CBCL, as compared with high grade CBCL, the intensity of CXCR5  [ **BLR1** ]  expression on neoplastic CD22 cells was lower than that of BCA 1  [ **CXCL13** ] .  Ref: 12855271 Eur J Cancer, 2003
- Memory but not naive T cells from tonsils are CXCR5  [ **BLR1** ]  and migrate in response to the B cell attracting chemokine 1  [ **CXCL13** ]  BCA 1  [ **CXCL13** ] , which is selectively expressed by reticular cells and blood vessels within B cell follicles.  Ref: 11104798 J Exp Med, 2000
- B cell attracting chemokine 1  [ **CXCL13** ] , a human CXC chemokine expressed in lymphoid tissues, selectively attracts B lymphocytes via  **BLR1**  CXCR5  [ **BLR1** ] .  Ref: 9463416 J Exp Med, 1998
- Here we show that DC that have migrated out of skin ex vivo migratory DC, migDC express 50 fold more CXCR5  [ **BLR1** ]  mRNA than fresh Langerhans cells and migrate in response to B lymphocyte chemoattractant  [ **CXCL13** ]  BLC  [ **CXCL13** ]  in vitro.  Ref: 11069061 Eur J Immunol, 2000
- In analogous fashion to certain Ag activated T and B cells, CR Fc DCs, expressing CXCR5  [ **BLR1** ] , localize to primary lymphoid follicles in response to CXC ligand 13 B lymphocyte chemoattractant  [ **CXCL13** ] .  Ref: 11994465 J Immunol, 2002
- BCA 1  [ **CXCL13** ]  and CXCR5  [ **BLR1** ]  were constantly coexpressed by CD22 B cells, while CD35 follicular dendritic cells coexpressed BCA 1  [ **CXCL13** ]  in PCBCL cells only.  Ref: 12855271 Eur J Cancer, 2003
- The chemokine receptor  **BLR1**  Burkitt s lymphoma receptor 1 CXCR5  [ **BLR1** ]  expressed by all mature B cells has to date been identified as the only known receptor for BCA 1  [ **CXCL13** ] .  Ref: 11554781 Cytokine, 2001
- Both Bordetella pertussis toxin and neutralizing anti CXCR3 anti CXCR5  [ **BLR1** ]  monoclonal antibodies block CXCL10 and  **CXCL13**  induction, respectively.  Ref: 12447991 J Cell Physiol, 2003
- Anti CXCR5  [ **BLR1** ]  monoclonal antibody and a ligand for CXCR5  [ **BLR1** ] , BCA 1  [ **CXCL13** ] , inhibited HIV 2 infection to NP 2 CD4 CXCR5  [ **BLR1** ]  cells.  Ref: 10600598 Virology, 1999
- At the same time, they downregulate CXCR5  [ **BLR1** ]  and CCR7 and have reduced responsiveness to the B and T zone chemokines  **CXCL13** , CCL19, and CCL21.  Ref: 11435471 J Exp Med, 2001
- BCA 1  [ **CXCL13** ]  was also a highly effective attractant for human blood B lymphocytes which express  **BLR1** , but was inactive on freshly isolated or IL 2 stimulated T lymphocytes, monocytes, and neutrophils.  Ref: 9463416 J Exp Med, 1998
- We show that BLC  [ **CXCL13** ]  stimulates calcium influx into, and chemotaxis of, cells transfected with BLR 1  [ **BLR1** ] .  Ref: 9486651 Nature, 1998

**Non-interactive relationship** (e.g. studied together, co-existance, homology, etc.)

- High level expression of BCA 1  [ **CXCL13** ]  and its receptor, CXCR5  [ **BLR1** ] , was observed in all mucosal lymphoid aggregates and in the mantle zone of all secondary lymphoid follicles in Hp induced gastric mucosa associated lymphoid tissue MALT.  Ref: 10562310 J Clin Invest, 1999
- We propose that activated skin DC utilize CXCR5  [ **BLR1** ]  and BLC  [ **CXCL13** ]  as a possible mechanism to home to B cell zones of LN, where they may have direct effects on B cells.  Ref: 11069061 Eur J Immunol, 2000
- **CXCL13**  expression was primarily observed in epithelial cells in acini and ducts of inflamed glands while its receptor, CXCR5  [ **BLR1** ]  BLR 1  [ **BLR1** ] , was expressed on the infiltrating mononuclear cells.  Ref: 11967114 Scand J Immunol, 2002
- In transfected cells, BCA 1  [ **CXCL13** ]  induced chemotaxis and Ca2 mobilization demonstrating that it acts via  **BLR1** .  Ref: 9463416 J Exp Med, 1998
- CXCR5  [ **BLR1** ] , the receptor for B lymphocyte chemoattractant  [ **CXCL13** ]  BLC  [ **CXCL13** ] , is required for normal development of Peyer s patches, inguinal lymph nodes, and splenic follicles.  Ref: 10843380 Immunity, 2000
- Identification of  **CXCL13**  and CXCR5  [ **BLR1** ]  in salivary glands suggests that the target organ plays an essential role in the inflammatory process by recruiting B and T cells.  Ref: 11967114 Scand J Immunol, 2002
- Reverse transcriptase polymerase chain reaction was used to detect chemokine transcripts of CXCR4, CXCR5  [ **BLR1** ] , BLC  [ **CXCL13** ] , and SDF 1 in PIOL and retinal pigment epithelium RPE cells after microdissection either by laser capture Arcturus or by manual dissection from frozen sections.  Ref: 12578791 Ophthalmology, 2003
- Here we show that CXC chemokine receptor CXCR 5  [ **BLR1** ] , the receptor for B lymphocyte chemoattractant  [ **CXCL13** ]  BLC  [ **CXCL13** ] , is upregulated on antigen specific CD4 T cells in vivo when animals are immunized under conditions that promote T cell migration to follicles.  Ref: 10523610 J Exp Med, 1999
- In these cells, as in human B lymphocytes expressing CXCR5  [ **BLR1** ] , BCA 1  [ **CXCL13** ]  does not induce a calcium flux.  Ref: 11554781 Cytokine, 2001
- Using gene targeted mice, we establish that B lymphocyte chemoattractant  [ **CXCL13** ]  BLC  [ **CXCL13** ]  BCA1  [ **CXCL13** ]  and its receptor, CXCR5  [ **BLR1** ] , are needed for B cell homing to follicles in lymph nodes as well as in spleen.  Ref: 10917533 Nature, 2000
- B cell attracting chemokine 1  [ **CXCL13** ]   **CXCL13**  and its receptor CXCR5  [ **BLR1** ]  are expressed in normal and aberrant gut associated lymphoid tissue.  Ref: 12171958 Gut, 2002
- Strong expression of BCA 1  [ **CXCL13** ]  and CXCR5  [ **BLR1** ]  was also detected in the transformed B cells of gastric MALT lymphomas.  Ref: 10562310 J Clin Invest, 1999
- Reciprocally, overexpression of CXCR5  [ **BLR1** ] , the receptor for the B zone chemokine  **CXCL13** , is sufficient to overcome antigen induced B cell movement to the T zone.  Ref: 11882900 Nature, 2002
- CONCLUSIONS BCA 1  [ **CXCL13** ]  and CXCR5  [ **BLR1** ]  are expressed in normal GALT structures as well as in irregular lymphoid aggregates in UC.  Ref: 12171958 Gut, 2002
- By contrast, murine bone marrow derived DC display 14 fold less CXCR5  [ **BLR1** ] , do not migrate to BLC  [ **CXCL13** ]  in vitro, and migrate strictly to T cell zones in LN.  Ref: 11069061 Eur J Immunol, 2000
- We analysed the immunophenotypic and molecular expression of BCA 1  [ **CXCL13** ]  B cell specific chemokine and CXCR5  [ **BLR1** ]  BCA 1  [ **CXCL13** ]  receptor in normal skin and different cutaneous lymphoproliferative disorders cutaneous T cell lymphoma CTCL.  Ref: 12855271 Eur J Cancer, 2003
- CD57 CXCR5  [ **BLR1** ]  T cells, hereafter termed germinal center Th GC Th cells, are localized only in GCs, lack CCR7, and are highly responsive to the follicular chemokine B lymphocyte chemoattractant  [ **CXCL13** ]  but not to the T cell zone EBI1 ligand chemokine.  Ref: 11413192 J Exp Med, 2001
- The chemokine chemokine receptor pair  **CXCL13**  CXCR5  [ **BLR1** ]  is required for the architectural organization of B cells within lymphoid follicles.  Ref: 12406091 Br J Haematol, 2002
- These results indicate that both CXCR3 CXCL10 and CXCR5  [ **BLR1** ]   **CXCL13**  receptor ligand pairs may play an important role in OB activity through the specific up regulation of two enzymes, which are involved in the bone remodeling process.  Ref: 12447991 J Cell Physiol, 2003
- During the extrafollicular response, plasmablast expression of CXCR5  [ **BLR1** ]  and responsiveness to B lymphocyte chemoattractant  [ **CXCL13** ]  CXCR5  [ **BLR1** ]  as well as to secondary lymphoid tissue chemokine CCR7 and stromal cell derived factor SDF 1 CXCR4 are lost while a weak response towards the CCR6 chemokine LARC is maintained.  Ref: 11180126 Eur J Immunol, 2001
- BCA 1  [ **CXCL13** ]  and CXCR5  [ **BLR1** ]  were constantly expressed in CBCL and PCBCL, but not in normal skin and CTCL.  Ref: 12855271 Eur J Cancer, 2003
- Together with the observed disturbance of B cell colonization in  **BLR1**  CXCR5  [ **BLR1** ]  deficient mice, the present results indicate that chemotactic recruitment by locally produced BCA 1  [ **CXCL13** ]  is important for the development of B cell areas of secondary lymphoid tissues.  Ref: 9463416 J Exp Med, 1998
- These findings indicate that LTalpha1beta2 and TNF have a role upstream of BLC  [ **CXCL13** ]  CXCR5  [ **BLR1** ]  in the process of follicle formation.  Ref: 9892622 J Exp Med, 1999
- We show here that CXCR5  [ **BLR1** ]   **CXCL13**  signaling activates alpha4beta1 integrin on CD4 CD3 cells.  Ref: 12354388 Immunity, 2002
- As the loss of the  **BLR1**  CXCR5  [ **BLR1** ]  receptor is sufficient to disrupt organization of follicles in spleen and Peyer s patches, BCA 1  [ **CXCL13** ]  may act as a B cell homing chemokine.  Ref: 11554781 Cytokine, 2001
- Our results indicate that BLC  [ **CXCL13** ]  functions as a BLR 1  [ **BLR1** ]  ligand and may guide B lymphocytes to follicles in secondary lymphoid organs.  Ref: 9486651 Nature, 1998
- Functional assays to evaluate CXCR3 and CXCR5  [ **BLR1** ]  demonstrated that their ligands CXCL10 and  **CXCL13** , respectively significantly induce the release of beta N acetylhexosaminidase, an enzyme involved in endochondral ossification and bone remodeling able to degrade important extracellular matrix components.  Ref: 12447991 J Cell Physiol, 2003
- Tumor cells stained positively for CXCR5  [ **BLR1** ] , the primary receptor for BCA 1  [ **CXCL13** ] .  Ref: 12393412 Blood, 2003
- **CXCL13** , SCYB13  [ **CXCL13** ]  and stromal cell derived factor 1 SDF 1, CXCL12, SCYB12 are chemotactic for human B cells, and their ligands CXCR4 and CXCR5  [ **BLR1** ]  are differentially expressed on B cells, including malignant B cells.  Ref: 12578791 Ophthalmology, 2003
- Deficiency in CXC chemokine receptor 5 CXCR5  [ **BLR1** ] , a receptor for B lymphocyte chemoattractant  [ **CXCL13** ]  BLC  [ **CXCL13** ] , also causes loss of splenic follicles.  Ref: 9892622 J Exp Med, 1999
- The sections were evaluated using immunohistochemistry avidin biotin complex immunoperoxidase technique for CXCR4, CXCR5  [ **BLR1** ] , BLC  [ **CXCL13** ] , and SDF 1 to detect the expression and location.  Ref: 12578791 Ophthalmology, 2003
- Follicular B helper T T FH cells make up a CD4 memory T cell subset with unique migration properties, defined by the expression of the chemokine receptor CXCR5  [ **BLR1** ]  and responsiveness to the CXCR5  [ **BLR1** ]  selective chemokine BCA 1  [ **CXCL13** ] , which is produced in B cell follicles.  Ref: 12527215 Immunol Lett, 2003
- Expression of chemokine receptors, CXCR4 and CXCR5  [ **BLR1** ] , and chemokines, BLC  [ **CXCL13** ]  and SDF 1, in the eyes of patients with primary intraocular lymphoma.  Ref: 12578791 Ophthalmology, 2003
